# Selective Attention in Early Word Learning: An Eye‐Tracking Study on Viewing Naturalistic Egocentric Scenes

**DOI:** 10.1111/infa.70043

**Published:** 2025-08-20

**Authors:** Yayun Zhang, Chen Yu

**Affiliations:** ^1^ Max Planck Institute for Psycholinguistics Nijmegen the Netherlands; ^2^ The University of Texas at Austin Austin Texas USA

**Keywords:** infant eye tracking, perception, referential uncertainty, visual attention, word learning

## Abstract

To learn a word from an everyday context, infants need to be able to link the heard word with the correct object perceived. A prevailing view of the early learning environment is that infants' world is bombarded with objects and words. Therefore, it is difficult to find the named object from many possible candidates. However, building correct word‐referent mappings relies on in‐moment visual selection, it is not clear what infants attend to when learning words in a naturalistic context. Toward this goal, we conducted an eye‐tracking experiment in which 12‐month‐old infants were presented with complex visual scenes extracted from infants' egocentric videos recorded during naturalistic parent‐child toy play. These scenes were selected at naming moments when parents labeled a toy object during free‐flowing play. We selected visual scenes from a mix of more or less ambiguous naming events that contained different visual properties of the named objects and measured infants' real‐time object‐looking behaviors. We found that, despite the different visual properties of infants' egocentric scenes, early visual attention is both selective and variable. Selective visual attention is highly constrained by the visual saliency of the learning scenes, but not influenced by labels or existing word knowledge. Infants are more likely to attend to the named object when it is salient in the egocentric view. Our results suggest that although infants' naturalistic learning environment appears to be messy in terms of the number of possible objects for a heard object name, their selective attention significantly reduces the in‐moment uncertainty associated with object name learning.

## 1 Introduction

Infants demonstrate knowledge of words as early as 6 months of age (Bergelson and Swingley 2012). By age two, they can already produce approximately over 50 words and continue to learn new words daily at a fast rate (Fenson et al. 1994). A large number of those words acquired in the first 2 years are object names (Goldin‐Meadow et al. 1976). To learn object names, infants need to associate an object label with its correct referent from the environment. However, because their early environment contains a lot of objects, happenings, and properties, the visual scene at any naming moment can be highly ambiguous, containing many potential referents of the heard word (Medina et al. 2011). This ambiguity problem faced by infant learners is termed *referential uncertainty* (Quine 1960).

We focused on object name learning not only because a majority of early acquired words are object names but also because early learned object names form the first building blocks of infants' language (Golinkoff et al. 1994). Many theories of early object name learning have been developed based on the assumption that infants need to infer the right referent from many word‐to‐world mappings available when they encounter a new word (i.e., Baldwin 1991; Booth and Waxman 2003; Jaswal and Markman 2001; Medina et al. 2011; Tomasello and Akhtar 1995; Yu and Smith 2007). However, it is not clear how much information infants select at the moment of hearing a label. If infants selectively attend to only one or a small number of objects, it would change the word learning problem infants encounter and subsequently alter the question researchers should investigate to understand early word learning. Thus, it is crucial to examine the attentional mechanisms that drive infants' in‐the‐moment selective attention when hearing object labels, as this greatly impacts what information they process to build word‐to‐world mappings.

### 1.1 Visual Attention and Word Learning

In developmental research, there is a long history of linking infants' attention with language development. When hearing speech and linguistic stimuli, infants' looking behavior is driven by their language knowledge. For example, Golinkoff et al. (1987) pioneered the *Preferential Looking* method to investigate infants' comprehension of nouns and verbs and found that infants who had not begun to produce any verbs yet looked significantly longer at and oriented faster to the objects and actions that match the heard sentence than the display that did not match, providing empirical evidence on their comprehension of both nouns and verbs. Fernald et al. (1998) used the *Looking‐While‐Listening* paradigm to measure how fast infants move their eyes to the picture of a known word as a measure of real‐time speech processing. They found that older children with more experience listening to language become faster in directing their gaze to the correct referent.

More recently, advances in eye tracking technology allow researchers to measure the dynamics of infants' eye movements with high resolution and precision, providing a window to reveal infants' real‐time information selection process. This technological advance has been utilized in various word‐learning tasks to investigate how learners resolve the referential uncertainty problem through a sequence of individually ambiguous learning situations (i.e., Yu et al. 2012; Trueswell et al. 2013). For example, Yu and Smith (2011) found that 14‐month‐old infants strategically sample statistical information when presented with multiple novel objects in a word learning task. The differences in looking patterns found between strong and weak statistical learners suggest that word learning is tightly linked to the momentary dynamics of attention. More generally, the study demonstrated that eye movement measures can provide insights into the mechanism through which infants accumulate statistical information over the course of word learning and real‐time looking behaviors predict later learning outcomes.

Most eye‐tracking studies on infant visual attention and language learning, such as those described above, measure learners' attention when viewing simple visual scenes, typically composed of a small number of objects spatially separated on a clean background (Colombo 2001; Yu and Smith 2011). Those simple visual scenes created for laboratory experiments are hardly representative of the complex visual scenes that infants encounter in the real world, which are typically cluttered with numerous objects and events. To understand how infants process visual information in the real world, more complex visual stimuli, such as natural scene pictures (Amso et al. 2014; van Renswoude, van den Berg, et al. 2019; Pomaranski et al. 2021; Oakes et al. 2024; Wass and Smith 2014) and children's television programs (Frank et al. 2009; Franchak et al. 2016) have been used in infant eye‐tracking experiments. These studies have shown that the physical salience of the stimuli plays a significant role in infants' attention allocation. Although real‐world pictures and films are more ecologically valid than highly simplified stimuli, they are still stimuli selected and created by adults, which differ significantly from infants' everyday visual experiences of the real world (van Renswoude, Visser, et al. 2019). Therefore, there is a need to study how the early visual sampling process is implemented through infants' self‐generated data with unique visual properties (Slone et al. 2019).

### 1.2 Word Learning From the Infant's Egocentric View

Recently, there has been a growing interest in documenting children's everyday visual experiences from their own point of view. Researchers mounted miniature cameras on the infant's head to record visual information perceived from the infant's perspective when they freely explore their environment (i.e., Bergelson et al. 2019; Franchak et al. 2011; Smith et al. 2011; Smith et al. 2015; Suanda et al. 2019; Sullivan et al. 2020; Long et al. 2024). This line of research has revealed that the visual scenes perceived by infants are dynamic, rapidly changing from moment to moment, as opposed to photos and videos taken from a third‐person perspective (i.e., Aslin 2009; Yoshida and Smith 2008; Yurovsky et al. 2013) or the view from a parent in the same environment (Smith et al. 2011; Bambach et al. 2018). This is because active infants constantly move their bodies to create visual information for underlying visuomotor processes to serve their ongoing actions and goals (Luo and Franchak 2020).

Even though the head‐camera approach records what is visually present in infants' egocentric view, it does not show what infants visually attend to from such a view. A recent study using head‐mounted eye tracking during toy play found that infant gaze data show a bimodal distribution—when hearing object labels, infant do not visually explore many objects present in their view, but rather select one single referent in view. The selected referent at each naming moment is equally likely to be the correct target or incorrect distractor (Yu et al. 2021). The unexpected finding from this observational study highlighted the critical role of visual attention in early word learning, which is further examined experimentally in the present study.

### 1.3 Current Study

While Yu et al. (2021) revealed that infants often fixate on a single referent—correct or incorrect—during parent labeling moments, those findings were based on naturally occurring, free‐flowing interactions where each infant experienced a unique visual environment. In contrast, our study introduces a complementary experimental approach that enables systematic investigation of how infants allocate attention when viewing the *same* set of naturalistic egocentric scenes. Specifically, the present study collected new datasets using a two‐step approach. In Step 1, we collected egocentric videos of infants playing with toys with their parents. From those toy‐play sessions, we carefully selected a set of egocentric scenes at the moments when parents label the toys. Past research has shown that egocentric scenes vary with different visual compositions (Bambach et al. 2018; Slone et al. 2019; Cain et al. 2025). For example, Bambach et al. (2018) demonstrated that the objects present in the visual field—and their relative size, salience, and position—shift dynamically based on whether a person is reaching, manipulating, or simply observing. Similarly, Slone et al. (2019) found that infants' egocentric views during play are not only structured by their own actions but also shaped by the behaviors and proximity of social partners, such as caregivers. Notably, the orientation of objects in the infant's visual field was found to be heavily influenced by both the infant's own manipulation of objects and the actions of their caregivers, emphasizing how social and self‐directed behaviors jointly shape egocentric scenes. These studies underscore that egocentric input is highly variable and context‐sensitive. Therefore, to closely approximate naturalistic learning situations, different types of egocentric scenes with varying visual properties were chosen to create different experimental conditions used in a subsequent free‐viewing task (see Section 2.3 for details). In Step 2, we displayed these scenes with different visual compositions on a computer screen to a group of infants and measured their visual selection by tracking their eye movements.

The two‐step hybrid approach was initially developed by Aslin (2009), which provides three methodological strengths. First, unlike previous experimental studies that created tightly controlled artificial stimuli, using egocentric visual scenes approximates the visual and contextual complexity that children encounter in real life. Second, in contrast to previous observational studies that used head‐mounted eye tracking during free‐flowing play, infants in the present study saw the same set of egocentric scenes. Holding the visual input constant across participants allowed us to compare whether individual infants deploy their gaze to select information in a similar or different manner. Third, experimentally manipulating scene types allowed us to disentangle the impacts of scene properties on referential selection, extending previous observational work to provide a more generalizable account of how visual attention supports word learning in infancy.

**FIGURE 1:**
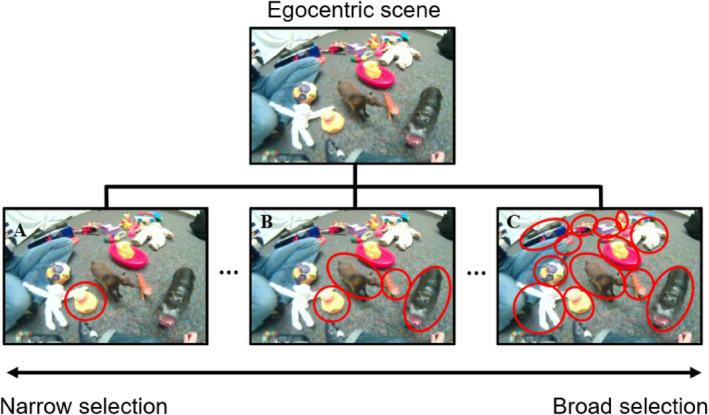
Top: An example of an egocentric scene where the parent labels the toy “hippo.” With the same egocentric scene (A–C), infants may selectively attend to a single object (A), some of the objects (B), or broadly attend to all of them (C).

Two experimental conditions were created using this paradigm. In condition 1, infants were shown a set of egocentric scenes while hearing an object labeling sentence for each scene, mimicking a word learning moment in naturalistic toy play. We aimed to address two questions on visual attention: (1) *Selectivity*—how broadly did infants sample objects in egocentric scenes? Given many objects in view to which the infant could potentially attend (Figure 1 top panel), one hypothesis is that infants might attend to only one (Figure 1A) or very few (Figure 1B) objects in view. Alternatively, they might attend to every object in view and register all the word‐referent mappings for word learning (Figure 1C). To answer this question, we measured how many objects infants attended to from all objects in view and how much attention they allocated to each attended object. (2) *Variability*—were different infants choosing the same object(s) to attend? If infants' attention is largely driven by the bottom‐up visual properties of a scene, then they would be attending to the same set of objects as they viewed the same scenes with the same visual properties. However, if different infants chose different objects to attend to, that could suggest that top‐down factors beyond just external stimuli drove infant attention. The variability analysis was intended to characterize the extent to which infant attention patterns were consistent across individuals. This allowed us to assess whether shared bottom‐up visual features alone could account for gaze behavior, or whether additional top‐down factors might also play a role. Regardless of the outcome of this analysis, we proceeded to examine how other factors such as labeling and prior knowledge influenced infants' attention, as these were central to our research questions.

In condition 2, the same egocentric scenes in condition 1 were shown but without labeling sentences during free viewing. The gaze data from conditions 1 and 2 were analyzed together to experimentally test three factors that may influence infant attention: (1) *Label effect*—are infants more likely to look at the target object after hearing its label? (2) *Prior knowledge effect*—if infants already knew the heard label, are they more likely to attend to it? (3) *Visual property effect*—how do different visual properties of the target object drive infants' attention to it? If infant attention is primarily driven by bottom‐up factors, their attention would be more scattered when viewing complex scenes containing many objects compared with scenes with fewer objects. If infant attention is mainly driven by top‐down factors, such as prior knowledge about objects and their labels, they would consistently choose to attend to certain objects based on their prior knowledge, regardless of scene compositions.

Quantifying the information that infants actively select and the factors influencing their visual selection will shed new insights into our understanding of early word learning in at least two critical ways. First, it will help us precisely define the word‐learning problem that infants face in everyday interactions, using learning input from their own perspective. Second, it will examine visual selection as a potential source of individual differences in early language learning.

## 2 Methods

This research was conducted in accordance with the guidelines outlined in the Declaration of Helsinki, and written informed consent was obtained from a parent or guardian for each child prior to data collection. All procedures in this study were approved by the Human Subjects and Institutional Review Boards at Indiana University (protocol no. 0808000094). All families were recruited from Bloomington, Indiana, a primarily white, non‐Hispanic community of working‐ and middle‐class families in the Midwest of the United States. The sample was broadly representative of Monroe County, Indiana (84% European American, 5% African American, 5% Asian American, 2% Latino, 4% Other) and consisted of predominantly working‐ and middle‐class families.

### 2.1 Collection of Egocentric Videos

Infants (*n* = 17, 9 boys), aged between 11.5 and 12.5 months, wore head‐mounted cameras during 10 min of toy play with their parents. We focused on 12‐month‐old infants because this age marks key developmental milestones that make it particularly well‐suited for studying visual attention in the context of word learning. By 12 months, infants begin to link visual objects with verbal labels—a foundational ability for vocabulary development. They also exhibit near‐adult‐like visual scene scanning and are developing “top‐down” attentional control, allowing them to selectively attend to relevant aspects of their environment (Oakes 2023). These emerging skills make 12 months a critical window for investigating how attention supports the early stages of language acquisition.

Play occurred in a 3‐m by 4‐m room with an assortment of 24 toys that were haphazardly placed on the carpeted floor before the start of the experiment. All toys sampled were commercially available ones (e.g., animals, vehicles) that children at this age commonly play with. All toy objects were small enough for infants to manipulate manually.

During play, all infants wore headgear (hat or headband) fixed with a small lightweight head camera set low on the child's forehead (Borjon et al. 2018). The head camera was from Watec manufacturer and had a 90‐degree diagonal field of view and a 30 Hz recording rate. After the infants were fitted with the camera, the parent was instructed to play with their infant as they would naturally at home, and then the experimenter left the playroom for the parent and infant to play alone for 10 min. In total, 170 min of head camera videos were collected.

### 2.2 Selection of Stimuli

#### 2.2.1 Visual Stimuli

To select scenes broadly representative of the infant's naturalistic visual environment during word learning, we first transcribed parent speech and identified those spoken utterances containing toy names (e.g., “Where is the duck?”). From these naming utterances, we targeted 11 out of 24 toys with the highest naming frequencies. These toys were: ball, doll, tiger, duck, hippo, giraffe, spinning top, Mickey Mouse, horse, telephone, and cow. We extracted egocentric video clips centered on parent naming of these toy referents to examine how infants attend to them in real time. These moments, when objects are explicitly labeled, are crucial for understanding the visual and attentional mechanisms that support word learning during everyday interactions.

**FIGURE 2:**
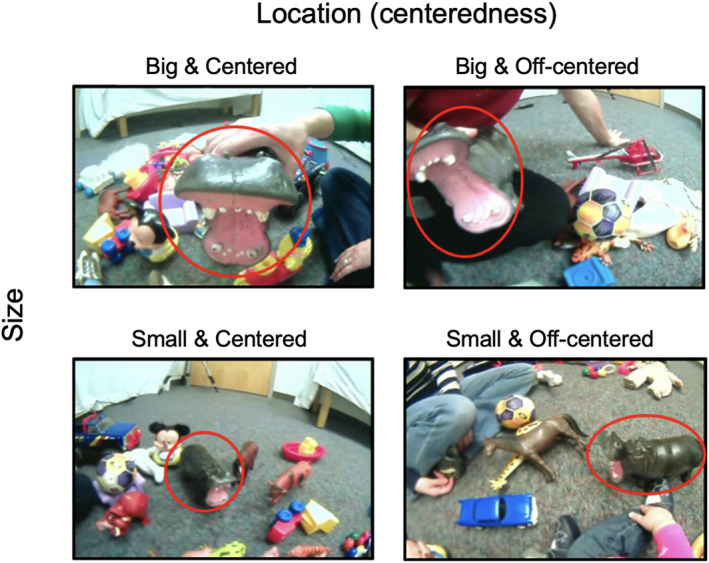
The four egocentric scenes featuring the target object “hippo” were used in the free‐viewing task. These scenes represent different types of learning scenes that infants may encounter during everyday word learning. Red circles around the target object were added for illustration purposes only and were not shown during the experiments.

Previous work using head‐mounted cameras shows that egocentric scenes from a child's view vary with different visual properties (Bambach et al. 2018; Slone et al. 2019; Cain et al. 2025). Accordingly, we used two visual properties—namely the size and location of the named objects, to choose representative scenes. For each of the 11 objects, a trained coder carefully chose four egocentric scenes varying in both visual size (big vs. small) and object location (center vs. off‐center) of the named objects (Figure 2) to create four types of learning scenes: big & centered, big & off‐centered, small & centered and small & off‐centered. Four distinct egocentric learning scenes were selected for each of the 11 objects. In total, 44 naming instances were used in the following experiments.

As shown in Figure 2, the four selected scenes have distinct differences in visual complexity and uncertainty. Table 1 shows three quantitative measures of visual stimuli in the four types of learning scenes: (1) *Number of objects in view*: There are many visible objects in view to which infants could direct their attention. (2) *Target object size*: On average, the target object occupies about 20% of the entire scene when it is big in view compared to 5% when it is small in view. In the two scene types with small targets, the largest distractor is also quite small (6.8%). Therefore, there are no visually dominant objects in these two types of scenes. (3) *Target object location*: The location of a target object was measured as the distance between the target center and the center of an egocentric scene. This distance measure was normalized with the maximum distance defined as 1 when a target object was at the corner of the scene. With this measure, the mean distance is about 0.01 for the two centered scenes and about 0.21 for the two off‐centered scenes. Taken together, the four types of egocentric scenes represent different degrees of referential uncertainty encountered by young learners when hearing an object label. All 44 scenes are available on the Open Science Framework.

**TABLE 1:** Visual property details averaged across all 11 toys in each of the four types of scenes.

	Number of objects in view		Proportional object size		Distance to center
	Mean	Max	Target (%)	Largest distractor (%)	(Max = 1)
Big & centered	10	20	22.3	5.2	0.008
Big & off‐centered	11	20	16.3	8.7	0.124
Small & centered	14	16	4.7	6.8	0.013
Small & off‐centered	15	18	5.2	6.8	0.305

#### 2.2.2 Auditory Stimuli

**FIGURE 3:**
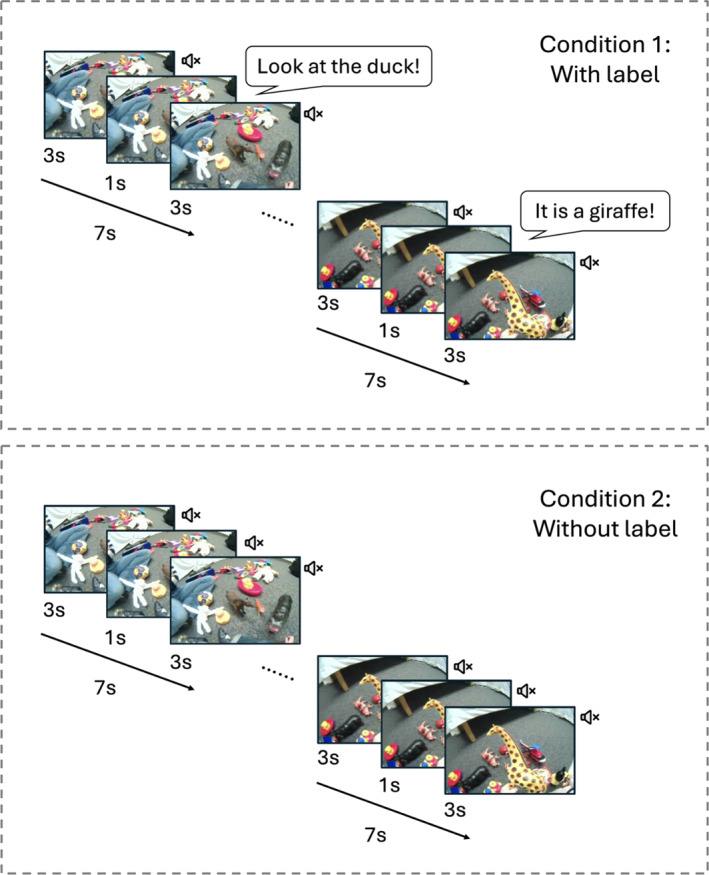
In the free‐viewing task, each egocentric scene was pseudorandomly shown for 7 s. There was a 3‐s silence before and after the labeling sentence in condition 1. The visual scenes were shown without labeling sentences in condition 2.

For condition 1, the audio from the original head‐cam videos was removed. To create a word learning context, each scene was accompanied by a labeling sentence. A female native English speaker recorded 44 infant‐directed labeling sentences. As shown in Figure 3, all labeling utterances were 1 s long, with the onset of the utterance occurring exactly in the middle of each 7‐s trial, so there were 3 s of silence both before and after the 1‐s labeling sentence. To keep infants attentive, the same object was labeled using different sentence structures, such as “Look at the __!”, “There is a __!”, “See the__!”, “It is a__!” and the same sentence structure never occurred consecutively. In condition 2, the same visual stimuli were used without labeling sentences.

### 2.3 Free‐Viewing Task

#### 2.3.1 Participants

Twenty‐three infants (11 girls) between 11.4 and 12.6 months of age (*M*_age_ = 12.20, SD_age_ = 0.31) and their parents participated in condition 1 and 26 infants (13 girls) between 11.5 and 12.8 months of age (*M*_age_ = 12.10, SD_age_ = 0.41) and their parents participated in condition 2. All infants received a gift at the end of the experiments for their participation. We determined our sample size by referencing prior studies with infant participants employing similar paradigms (i.e., van Renswoude, van den Berg, et al. 2019), which utilized comparable total trial numbers to maintain statistical power. Our repeated‐measures design, which involves multiple trials per subject with relatively long trial durations, further strengthens statistical power by reducing error variance and leveraging within‐subject correlations. Additionally, our multilevel modeling approach enabled us to include infants who completed only a small number of trials, accommodating the challenges of data collection in this population.

#### 2.3.2 Apparatus

Infants' eye gaze was measured by a Tobii 1750 eye tracker. The principle of this corneal reflection tracking technique is that an infrared light source is directed at the eye and the reflection of the light on the corneal relative to the center of the pupil is measured and used to estimate where the gaze is fixated. The eye‐tracking system recorded gaze data at 50 Hz (accuracy = 0.5°, and spatial resolution = 0.25°) as a viewer watched an integrated 17‐inch monitor with a resolution of 1280 × 1024 pixels. E‐prime software was used to present the stimuli and to automate the recording of eye location with the eye tracker software.

#### 2.3.3 Procedure

Infants were seated on their caregivers' laps approximately 60 cm from the monitor in a quiet room. Parents were instructed to keep their children seated, facing forward, and refrain from talking to them or directing their attention. Parent were also told to either look down or close their eyes throughout the entire procedure to avoid influencing their children's behavior.

The point of gaze was calibrated with a toy animation that appeared randomly at five locations (four corners and center) across the screen, one at a time. After successful calibration, the first trial began with the centered presentation of a laughing‐baby animation to orient infants' attention to the screen. The animation remained on the screen until the infants fixated at the center of the screen for 500 ms. This triggered the start of an experimental trial during which the selected egocentric scenes would be presented full screen. On each trial, one scene image was presented for 7 s, accompanied by a 1‐s labeling sentence presented in the middle of the trial for condition 1. In total, 44 scenes (11 toys × 4 scene types) were displayed. The temporal order of scenes was pseudorandomized so that scenes showing the same object and same scene type did not appear consecutively. The laughing‐baby attention‐grabbing slides were interspersed every 4 trials to maintain the infant's attention. While infants were attending to the screen, they also heard soft music in the background in both conditions. This is commonly used in infant eye‐tracking studies to keep the infants on task (Oakes and Ellis 2013). The entire testing session lasted about 6 min. After the study, parents completed the infant version of the MacArthur‐Bates Communicative Development Inventory (Infant‐MCDI), a parent questionnaire designed to assess children's receptive and productive vocabularies (Fenson et al. 1994).

### 2.4 Data Processing

**FIGURE 4:**
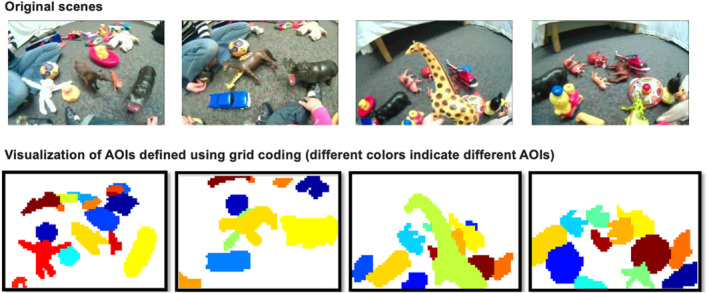
Sample images illustrating Area of Interest (AOI) defined via a grid‐based method. Each 480 × 720‐pixel image (top row) was divided into a 48 × 72 grid, with each grid cell covering a 10 × 10‐pixel area (bottom row). A trained coder annotated the object(s) present in each cell, enabling precise and efficient object separation comparable to manual outlining.

We first developed a grid‐based annotation system by dividing each 480 × 720‐pixel image into a 48 × 72 grid, where each grid covers a 10 × 10‐pixel area of the scene (Figure 4). A trained coder then carefully went through each grid and annotated the object present in each grid. For a grid that contains multiple objects, the largest object in the grid was assigned. With this grid‐based annotation, gazed objects were automatically calculated by superimposing infant gaze data on the annotated images. This approach improves upon traditional bounding box methods commonly used to annotate cluttered naturalistic scenes by providing greater accuracy, consistency, and objectivity. The output of this coding was a temporal stream of gaze data points ‐ 350 (50 Hz × 7 s) points per trial that indicate the attended objects over 7 s of the viewing window. The data are openly available on the Open Science Framework.

Although infants were generally attentive during the task, not all of them attended to all the trials. They sometimes looked away from the screen or moved their head out of the tracking area. Therefore, a trial was included only if the infants attended to the screen for at least half of the trial (3.5 s). Based on this standard, 612 out of 1012 trials in condition 1 were included with an average of 83% of gaze data points per trial (big and centered = 148 trials, big and off‐centered = 150 trials, small and centered = 150 trials, small and off‐centered = 164 trials). On average, each infant contributed 26.6 trials (min = 3 trials, max = 44 trials). With the same exclusion criteria applied to the data collected in condition 2, 503 trials were included in subsequent analyses (big and centered = 123 trials, big and off‐centered = 121 trials, small and centered = 121 trials, small and off‐centered = 138 trials). Each infant contributed an average of 20.1 usable trials, ranging from 2 to 43 trials. It is worth noting that there was no significant difference between the two conditions in terms of trials lost due to the infants' attention being diverted from the screen. Labeling did not distract or enhance children's overall attention to the task.

## 3 Results

### 3.1 Selectivity and Variability in Infant Attention

The results from condition 1 focus on infants' object‐looking behavior within the 7‐s viewing window, including both before and after an object label. This set of analyses aimed to examine how infants visually select available objects in view when hearing a labeling sentence. For our subsequent linear mixed‐effects model analyses, we employed a stepwise simplification protocol to determine the random effects structure for each model. Initially, we specified the maximal random‐effects structure, including random intercepts for subject, object, and trial number. When models failed to converge, we systematically simplified the random effects structure by sequentially removing random intercepts in the following order: trial number, object, and finally subject, re‐fitting the model after each removal. This approach ensured that we retained the most complex converging structure, balancing model complexity with convergence stability.

#### 3.1.1 Selectivity

**FIGURE 5:**
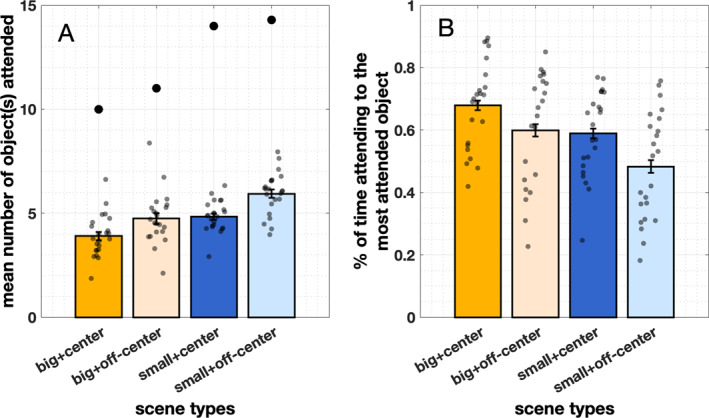
(A) Mean number of objects attended in the four scene types. The 4 large black dots represent the mean total number of objects in view for each scene type. (B) Proportion of time infants attend to the most attended object. Error bars represent ± standard error. Small gray dots represent individual data points (*n* = 23).

We first measured the total number of unique objects infants attended. Given more than 12 objects (*M* = 12.36, SD = 4.90) in view and a 7‐s naming window, infants only attended to a small subset of objects per trial (*M* = 4.92, SD *=* 0.62), which was fewer than half of the objects in view. In the most cluttered scene type with small and off‐centered target, infants attended only 6 out of 15 available objects (Figure 5A, *M*_big_centered_ = 3.90, SD_big_centered_ = .96; *M*_big_off‐centered_ = 4.74, SD_big_off‐centered_ = 1.26; *M*_small_centered_ = 4.83, SD_small_centered_ = 0.71; *M*_small_off‐centered_ = 5.94, SD_small_off‐centered_ = 0.97).

To evaluate how the fixed effects of target size, location, and the total number of objects in view jointly influenced infants' visual selectivity, we fitted a mixed‐effects model including all three predictors assuming a Poisson distribution appropriate for count data. Likelihood ratio tests comparing this full model (unique obj looked ∼ size + location + total number of obj + (1 | subj) + (1 | obj) + (1 | trial), family = Poisson) to reduced models excluding each predictor in turn revealed that both target location and the total number of objects significantly predicted the unique number of objects looked at, even when controlling for the other variables. Specifically, adding total number of objects to a model with size and location significantly improved model fit, *χ* ^2^ (1) = 56.89, *p* < 0.001, and adding location to a model with size and total objects also improved fit, *χ* ^2^ (1) = 6.46, *p* = 0.011. In contrast, adding size to a model with location and total objects did not significantly improve model fit, *χ* ^2^ (1) = 0.28, *p* = 0.59. These results suggest that infants' selective attention, in terms of the number of distinct objects attended, is influenced by the centerness of objects and the total number of objects in view. Target size does not affect how many objects infants choose to attend.

**FIGURE 6:**
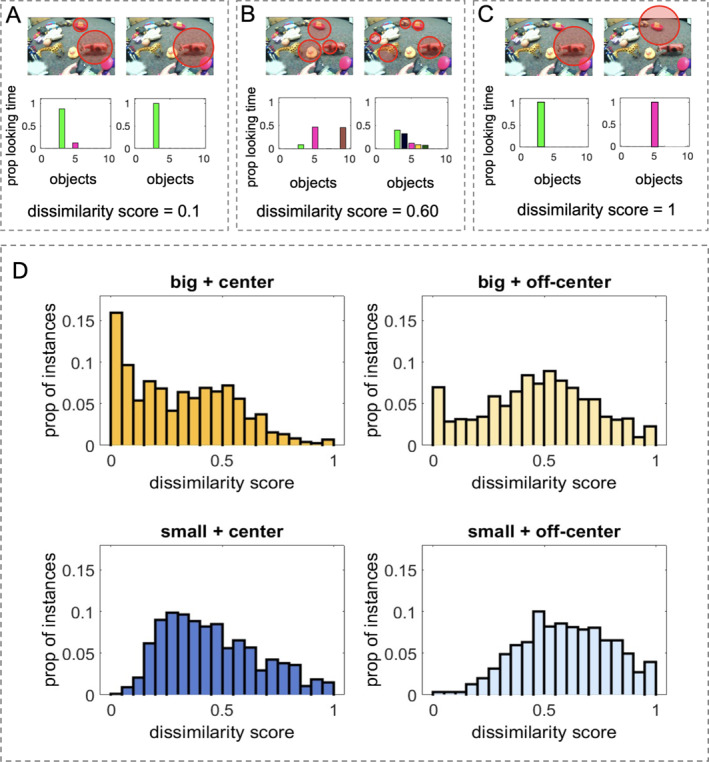
Variability among attended objects is measured by calculating pairwise differences of gaze distribution between any two infants, yielding a dissimilarity distance score between 0 and 1. While two similar‐looking distributions would yield a low dissimilarity score (A), two different‐looking distributions would yield a high dissimilarity score (C), partially overlapping gaze patterns, such as when infants look at some of the same objects but not others, would yield a moderate dissimilarity score (B). For each type of scenes, a histogram of the dissimilarity scores from all pairwise comparisons are shown in (D).

We further examined how much time infants allocated their attention among the selected objects. Did infants attend to those objects equally frequently or did they only primarily attend to one object? We measured the proportion of looking time at the most attended object and found that across all types of visual scenes, infants spent more than half of the time (*M* = 58.38%, SD = 5.16%) looking at one single object (*M*_big_centered_ = 67.91%, SD_big_centered_ = 7.41%; *M*_big_off‐centered_ = 59.88%, SD_big_off‐centered_ = 9.19%; *M*_small_centered_ = 58.97%, SD_small_centered_ = 7.40%; *M*_small_off‐centered_ = 48.30%, SD_small_off‐centered_ = 9.63%, Figure 6B). These patterns suggest that across different types of scenes, infants did not distribute their attention equally among the attended objects, but they tended to choose a single object over others and spend a significant amount of time attending to one object. We conducted similar mixed‐effects analyses to examine whether *target size, location*, and *total number of objects* predicted the proportion of time infants looked at the most attended object. We first assessed the distributional characteristics of the proportion looking time data and found that they were not normally distributed, even after standard transformations (e.g., log, square root). Given that the outcome is bounded between 0 and 1 and continuous, we opted to use a generalized linear mixed‐effects model with a beta distribution, implemented via the glmmTMB package. This approach is well‐suited for modeling proportion data and accounts for the bounded nature of the response variable. We adjusted the data to ensure it fell strictly within the (0, 1) interval (as required for beta regression). The full model included size, location, and total number of objects as fixed effects, (full model: max_look_obj ∼ size + location + total_obj, family = beta). Likelihood ratio tests comparing nested models showed that including total number of objects significantly improved model fit compared to a model without it (*χ* ^2^ (1) = 240.98, *p* < 0.001). Similarly, location also contributed significantly (*χ* ^2^ (1) = 4.61, *p* = 0.03), as did size (*χ* ^2^ (1) = 4.51, *p* = 0.03). These results indicate that all three predictors uniquely and significantly influence infants' proportional looking time toward the most attended object.

In summary, although some visual properties of scenes influenced infants' object selectivity, infants typically sampled only about half of the objects in view. Moreover, they did not treat all attended objects equally as candidate referents; instead, they devoted the majority of their attention to a single object for more than half of the viewing time. This consistent pattern of high selectivity across different scene compositions created learning situations in which referential uncertainty at the moment of labeling was markedly reduced.

#### 3.1.2 Variability

Given the selectivity in infants' visual attention, we next investigated which object(s) infants selected. More specifically, if different infants consistently selected the same subset of objects from a scene, their attention is likely to be driven by the shared external properties of the visual scene, such as object size, object location, and scene composition. Alternatively, if different infants chose to attend to different objects, this result would suggest that other internal factors beyond external stimuli may play a role in driving infants' selective attention.

To quantify consistency and variability across individual infants, we compared attention distribution over all available objects in a scene. Given any two infants, a pairwise comparison was made by calculating the absolute difference between the two gaze distributions. For example, if the two infants looked at the object cow 100% of the time, the dissimilarity score of this identical‐looking pattern is 0 (minimum dissimilarity score = (|1−1|)/2 = 0). If one infant looked at the cow 100% of the time, and the other infant looked at the cow 90% and another object 10% of the time (Figure 6A), those highly consistent looking patterns yield a low dissimilarity score (dissimilarity score = (|1−0.9| + |0−0.1|)/2 = 0.1). If the two infants chose different sets of objects to look at during an entire trial (Figure 6C), the two different‐looking patterns would yield a high dissimilarity score (maximum dissimilarity score = (|1−0| + |0−1|)/2 = 1). Thus, similar‐looking distributions would yield low dissimilarity scores (close to 0), whereas different‐looking distributions would yield high dissimilarity scores (close to 1; Figure 6B).

As shown in Figure 6D, we plotted the histograms of dissimilarity scores from each of the four scene types. A positively skewed distribution toward high dissimilarity suggests that infants as a group choose different objects to attend. A negatively skewed distribution toward low dissimilarity means that infants choose similar objects to attend to. We then quantified the skewness of each distribution and found that the big‐and‐centered scene type has the most positively skewed distribution (skewness = 0.98). The big‐ and‐off‐centered and the small‐center scene types generally approximate a normal distribution with skewness scores of 0.12 and 0.09 respectively. The small‐and‐off‐centered scene type is negatively skewed with a skewness score of −0.19.

We ran a series of Mann‐Whitney *U* tests comparing different distance distributions and found all pairwise comparisons to be significantly different (Table 2), suggesting that the degree of variability in dissimilarity scores varied depending on the scene type. Specifically, cluttered scenes—and in particular, those classified as *small‐and‐off‐centered*—elicited highly variable attention patterns across individual infants. In these scenes, multiple small objects were spatially distributed across the field of view. As a result, different infants appeared to select different subsets of objects to attend to, suggesting that internal factors such as prior experiences, personal preferences, or moment‐to‐moment fluctuations in attention may have played a larger role in guiding visual selection—rather than low‐level saliency alone, which would be expected to drive infants toward attending to the same highly salient features or objects.

**TABLE 2:** Results from Mann‐Whitney *U* test comparing visual sampling consistency between any two types of scenes.

	Big & centered	Big & off‐centered	Small & centered
Big & centered	—	—	—
Big & off‐centered	*Z* = 13.66, *p* < 0.001	—	—
Small & centered	*Z* = 13.17, *p* < 0.001	*Z* = 2.59, *p* < 0.01	—
Small & off‐centered	*Z* = 25.19, *p* < 0.001	*Z* = 11.69, *p* < 0.001	*Z* = 15.47, *p* < 0.001

In contrast, less cluttered scenes, especially those where a large object was positioned in the center (*big‐and‐centered* scenes), led to markedly more consistent patterns of visual attention. Here, infants were more likely to converge on the same object or region of interest. The visual properties of these scenes—such as a single salient object placed centrally—may have strongly guided infants' attention in a similar way across participants. These external cues appear to guide attention toward the same focal point and reducing variability in object selection. However, even in these cases, variability was not eliminated. For example, in *big‐and‐centered* scenes, approximately a quarter of the instances had dissimilarity scores exceeding 0.5, indicating that a substantial proportion of infants still attended to different objects despite the presence of a visually dominant referent. This suggests that while external structure can guide attention, internal factors continue to influence visual selection even in relatively simple scenes containing a centrally placed salient object.

Our selectivity and variability results suggest that despite viewing clustered scenes with many visible objects, infants tend to focus their attention on only a few objects when hearing a label. This high selectivity seems invariant across different types of visual scenes, suggesting infants' own visual sampling process is highly selective by nature. Moreover, our results also suggest that infants do not sample their environments uniformly; rather, their attention is dynamically influenced by the constraints of the visual input. This variability in attention—though more pronounced in cluttered scenes—is still present, albeit to a lesser extent, even in less cluttered scenes with a clearly dominant visual referent. These findings on selectivity and variability carry important implications for downstream processes like language learning, where consistent attention to labeled targets can influence how reliably infants map words to their referents. To build on this, we next examined how infants' visual sampling behavior might shape early word learning. The following analyses focus on infants' attention to labeled target objects and the factors that may guide or constrain it.

### 3.2 Infant Attention to Labeled Objects

Early word learning requires infant learners to link seen objects with heard words. Previous studies show that during toy play, infants' sustained attention to a labeled object during a labeling moment is a reliable predictor of their success in learning the association between the name and the toy object (Yu et al. 2019). The more infants attend to a labeled object, the more likely they will learn the label‐object mapping. Therefore, we used the proportion of time that infant attention was directed to the labeled object as a measure to quantify the accuracy of infants' visual sampling process. To experimentally compare the potential effects of labeling on infant attention, we used the data from two conditions, one with labeling (condition 1) and one without (condition 2) to examine the following three factors that may influence infant attention to target objects: (1) *Labeling effect*: Comparing infant attention with or without object labeling. (2) *Prior knowledge effect*: Comparing infant attention to objects with known versus unknown names. The Infant‐MCDI reports from the parents were used to measure which target names infants already know. (3) *Visual property effect*: Comparing infant attention across the four types of scenes with different target visual properties.

#### 3.2.1 Labeling Effect

We first examined whether hearing an object label drives infants' attention to the named target. We focused on two temporal windows within a trial: a three‐second window before the onset of labeling and a three‐second window after the offset of labeling. If labels drive infants' real‐time attention to the labeled object, their attention to the target should be similar before labeling between the two conditions but increase after hearing an object label in the labeling condition.

**FIGURE 7:**
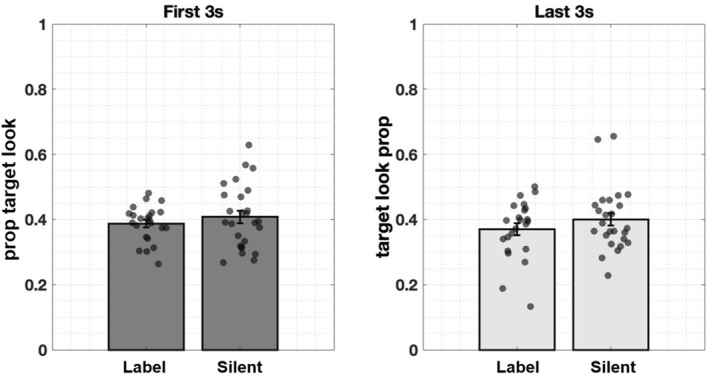
Infants' attention to target objects during the first and last 3 s of a trial in the two conditions. These timing windows correspond to 3 s before and 3 s after labeling in the condition 1. Infants' attention to target does not change across windows, suggesting that their target looking behaviors are not driven by labels. Error bars represent ± SE.

As shown in Figure 7, when presented with the same visual scenes, infants exhibited similar looking behaviors toward target objects, regardless of whether a label was heard or not (First 3s: *M*_label_ = 0.39, SD_label_ = 0.05; *M*_silent_ = 0.41, SD_silent_ = 0.10; Last 3s: *M*_label_ = 0.37, SD_label_ = 0.09; *M*_silent_ = 0.40, SD_silent_ = 0.10). To examine whether infants' attention to the target object differed between labeling and silent conditions—and whether this effect was modulated by object size or location—we ran two generalized linear mixed‐effects models. Each model predicted condition (label vs. silent) from the proportion of time infants looked at the target object during either the 3 s before or 3 s after labeling, including object size and location as fixed effects and a random intercept for subject (full model: condition ∼ prop_target_look + size + location + (1 | subj), family = binomial). As expected, in the pre‐label model using proportion of target look during the 3 s window before labeling, none of the predictors significantly improved the model fit when removed: proportion of target looking before the label (*χ* ^2^ (1) = 0.23, *p* = 0.64), object size (*χ* ^2^ (1) = 0.29, *p* = 0.59), or object location (*χ* ^2^ (1) = 0.44, *p* = 0.50). Interestingly, the post‐label model also showed no significant effects of proportion of target looking (*χ* ^2^ (1) = 0.06, *p* = 0.81), size (*χ* ^2^ (1) = 0.05, *p* = 0.82), or location (*χ* ^2^ (1) = 0.002, *p* = 0.96). These results suggest that infants' visual attention to the target object did not differ based on whether a label was provided, nor was it significantly influenced by the target object's size or location.

**FIGURE 8:**
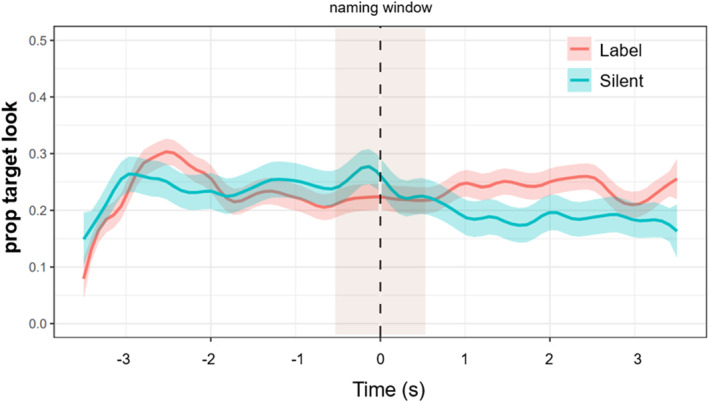
Temporal profile of the infant's visual attention to the target object within a 7‐s window (3 s before and after naming window). Attention to the target did not significantly differ before versus after labeling.

To further examine how gaze patterns changed in response to labeling, we generated temporal profiles estimating the likelihood that children were looking at the target object across a 7‐s trial window (3 s before and after the labeling sentence). Using moment‐by‐moment gaze data, we tracked shifts in attention relative to the 1‐s labeling event at the trial midpoint. If labeling influenced attention, we would expect to observe differences in gaze behavior between the label and silent conditions following the naming event. As shown in Figure 8, infants' gaze toward the target object was plotted for both conditions and no noticeable shifts in attention were observed following labeling.

To statistically compare these temporal profiles, we employed a permutation test designed for time‐course data (Abbot‐Smith et al. 2017). First, we computed a test statistic for each 100 ms time bin. Adjacent time bins with significant test statistics (*p* < 0.05) were grouped together, under the assumption that contiguous differences reflect a single underlying processing component. We then permuted the conditions (label vs. silent) to generate a null distribution representing the likelihood of observing our results by chance. Finally, we compared the observed cluster statistics to this null distribution to assess significance. No significant clusters were found when comparing infant target look in the label versus silent conditions across the 7‐s window. This suggests that labeling alone did not reliably shift children's attention toward the target object.

#### 3.2.2 Prior Knowledge Effect

Previous work has shown that 6‐to‐9‐months‐old infants fixate on the target object upon hearing a known name (Bergelson and Swingley 2012). Among the 11 selected toy objects, some infants may already know some of the object names. If so, we might observe an increase in attention to the known objects during the labeling moments.

To investigate whether prior knowledge drives visual attention, we divided all trials from the labeling condition into known and unknown trials based on Infant‐MCDI responses reported by parents. Nine of the 11 words we used in our study were on the Infant‐MCDI list. “Hippo” and “spinning top” were not included in the Infant‐MCDI, but since these two object names were likely too advanced for 12‐month‐old infants, we treated the hippo and spinning top trials as unknown for all infants. Based on this criteria, 34.5% (*n* = 211) of 612 trials were counted as known, and 65.5% (*n* = 401) were counted as unknown.

**FIGURE 9:**
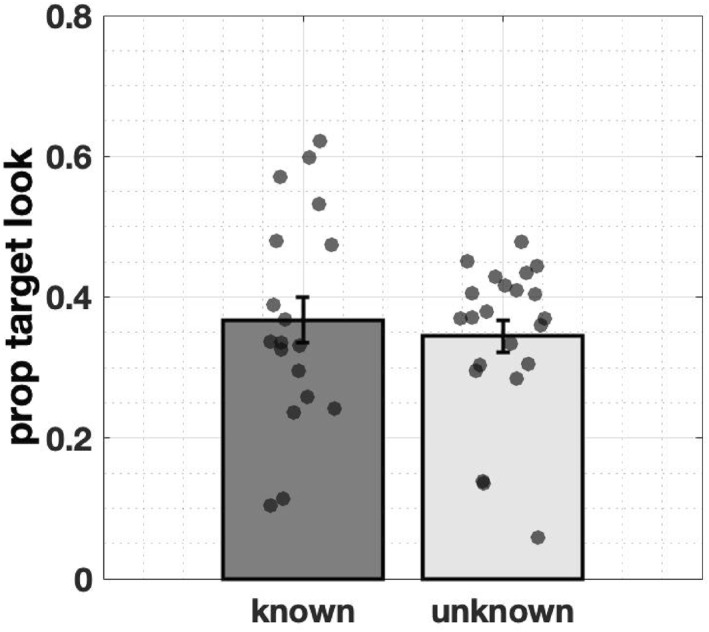
A comparison of target looks between known and unknown trials during the 3‐s window after labeling shows that infants' attention to the target does not differ between known and unknown trials. Error bars represent ± SE.

To measure the effect of prior knowledge, we focused on gaze data in the 3‐s window after hearing a label. As shown in Figure 8, there was no difference between known and unknown trials (*M*_known_ = 0.37, SD_unknown_ = 0.15; *M*_unknown_ = 0.34, SD_unknown_ = 0.11, Figure 9). To statistically test this, we fit a generalized linear mixed‐effects model with a binomial distribution and logit link, predicting word knowledge (known vs. unknown) from the proportion of time infants looked at the target object after labeling. The model included random intercepts for both subjects and objects (known ∼ prop_target_after_naming + (1 | subj) + (1 | obj), family = binomial). Results revealed no significant effect of post‐labeling looking time on word knowledge (*β* = 0.21, SE = 0.46, *z* = 0.47, *p* = 0.64), suggesting that visual attention after hearing a label was not associated with whether infants knew the labeled object. In other words, even when infants recognized an object name, they did not consistently look more or less at the corresponding object after hearing the label. One possible explanation is that egocentric scenes collected from toy play are much more cluttered than those created for well‐controlled experimental paradigms such as the looking‐and‐listening paradigm. Therefore, when viewing more cluttered scenes, infants' real‐time visual selection process is likely to be driven by multiple factors simultaneously, and prior knowledge alone may not be strong enough to push the infants to look more toward the target (Tummeltshammer et al. 2014).

#### 3.2.3 Visual Property Effect

Previous research has found that infants tend to attend to objects with visual saliency (Frank et al. 2014; van Renswoude, van den Berg, et al. 2019). In the four types of egocentric scenes, the target objects have different visual properties in terms of size and location (centeredness). Here, we compared gaze data in those four types to examine whether the visual properties of the target influence target‐looking time. Given no label effect and no prior knowledge effect, we combined data from conditions 1 and 2 and utilized gaze data from the entire 7‐s window to examine whether visual properties of the target influence target‐looking behaviors. A total of 1115 trials are included (big and centered = 271 trials, big and off‐centered = 271 trials, small and centered = 271 trials, small and off‐centered = 302 trials).

**FIGURE 10:**
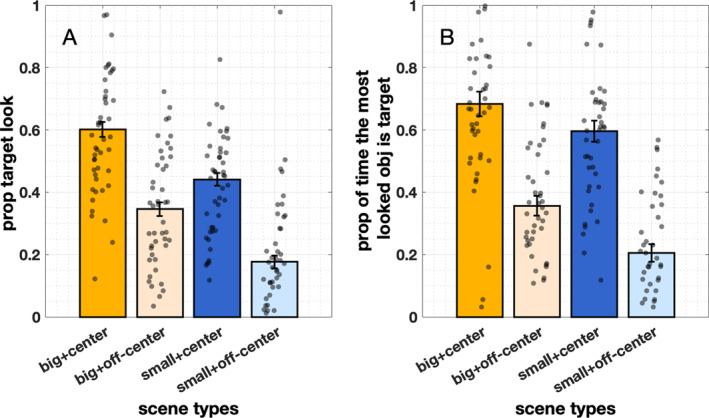
(A) Proportion of time looking at target objects. (B) Mean percentage of trials that the most attended object is the target object. Error bars represent ± SE. Gray dots represent individual data points (*n* = 49).

When the target object was big in view, infants looked at the target object more compared with the other two conditions wherein the target object was small (Figure 10A, *M*_big_ = 0.48, SD_big_ = 0.11; *M*_small_ = 0.31, SD_small_ = 0.15). Similarly, infants looked at the target more when it was centered in view than off‐centered in view (*M*_center_ = 0.52, SD_center_ = 0.11; *M*_off‐center_ = 0.27, SD_off‐center_ = 0.14). Likelihood ratio tests comparing full models (prop_target_look ∼ size + location, family = beta) to reduced models demonstrated that including size significantly improved model fit (*χ* ^2^ (1) = 154.57, *p* < 0.001), as did including centeredness *(χ* ^2^ (1) = 150.18, *p* < 0.001). These results suggest that both object size and centeredness significantly increase infants' attention to the target object. When the target was both large and centered, infants spent over 60% of their time looking at the visually salient target. We also conducted the same analyses using raw target looking time (max = 7 s)—recognizing that raw looking time captures absolute engagement, while proportion contextualizes that engagement relative to other regions, and both metrics are common in infant eye‐tracking research. We found that raw target looking time measured mirrored the proportion looking time findings (see Supporting Information S1: Figure 1).

Different accounts of early word learning (i.e., associative learning vs. hypothesis testing; see Yu and Smith 2012a; Zhang et al. 2019) agree that the most attended object after hearing its label is considered the top candidate for the label. To quantify the effects of infant attention on early word learning, we next calculated the percentage of trials in which the object that infants looked at most was the labeled target (most_looked_is_target ∼ location + size + (1 | subj), family = binomial). Consistent with our previous measures, we found that both size (*M*_big_ = 0.52, SD_big_ = 0.20; *M*_small_ = 0.40, SD_small_ = 0.21; *χ* ^2^ (1) = 37.04, *p* < 0.001) and location (*M*_centered_ = 0.63, SD_centered_ = 0.19; *M*_off‐centered_ = 0.28, SD_off‐centered_ = 0.20, *χ* ^2^ (1) = 99.41, *p* < 0.001) were significant predictors of whether the most attended object was the target (Figure 10B).

Taken together, the visual properties (i.e., size and location) of a target object significantly influence the likelihood that infants will select that object to attend to. If the target object is visually dominant in view, and the infants' attention is likely already drawn to it, then providing its label at that moment may increase the likelihood of successful word‐referent mapping. On the contrary, neither labeling itself nor prior knowledge drives infants' attention toward the target objects.

## 4 General Discussion

Previous studies with head‐mounted cameras provide useful insights on the visual information available from the infant's point of view (Yurovsky et al. 2013; Smith et al. 2015; Bergelson et al. 2019; Franchak et al. 2011; Sullivan et al. 2020; Long et al. 2024). However, little is known about the visual information that infants select from their egocentric view. The present study experimentally examined infants' moment‐by‐moment attention on egocentric visual scenes and found converging evidence supporting three major findings. First, infants' visual attention during parent naming moments is highly selective. Among all the visible objects in view, only a small subset of objects is made into the learners' perceptual system. Second, infants' visual system is not only selective, but also variable across different individuals. Different infants select different subsets of objects to attend to. The individual differences observed in visual selection may be a key factor contributing to individual differences in early word learning. Third, neither labeling nor prior knowledge seems to influence infants' visual selection. Instead, the composition of an egocentric scene and the visual properties of the target object in a scene play a critical role in attracting infants' attention to the correct target. Infants are significantly more likely to attend to a target object that is big and centered in view. Herein, we discuss the implications of these three main findings.

### 4.1 Selectivity and Variability Redefine the Word Learning Problem

It is commonly assumed that infants' early word learning environment is bombarded with many words and many objects (Medina et al. 2011). Although this *referential uncertainty* problem has been a focus of early word‐learning research for decades, little is known about the degree of uncertainty infants face and how infants actively select information to solve this problem. A common assumption is that infants are likely to distribute their attention more broadly when more information is available in a complex learning environment. However, our current findings suggest the opposite. Regardless of the complexity of egocentric scenes and the number of objects in view, infant selects a small subset of information for learning. In other words, the complexity of egocentric scenes does not change “how much” information is selected but “what” is selected.

For the “what” question, visual attention serves multiple cognitive functions in everyday activities. Young learners rely on vision to guide their actions (Gibson 1988), and visual attention acts as a filter, helping individuals select relevant stimuli and suppress distractions in complex environments (Desimone and Duncan 1995). Early in life, this attentional system plays a crucial role in shaping perception, memory, and learning. Bottom‐up attention is driven by salient visual features—such as color, motion, or brightness—processed in parallel across the visual field to generate a “saliency map” that guides eye movements toward the most prominent locations (Itti and Koch 2001). While bottom‐up mechanisms often dominate initial exploration, top‐down factors—such as internal goals, prior knowledge, or social cues—gradually exert more influence over visual selection (Desimone and Duncan 1995). In everyday contexts, visual attention flexibly shifts between these competing influences. At one moment, it may be guided by top‐down goals, such as searching for a favorite toy; at the next, it may be captured by bottom‐up saliency, such as a caregiver waving and saying goodbye. Given the dynamic and multifaceted nature of real‐world activities, the weighting of these factors can change moment to moment.

This may explain the highly variable visual selection patterns observed in the current study: different infants may prioritize different competing cues, leading them to attend to different subsets of available objects. In the current study, prior knowledge did not appear to have a direct influence on infants' visual attention. However, this does not mean that existing knowledge has no effect at all; rather, it may interact with other top‐down factors—such as transient internal goals and implicit task interpretations (e.g., infants forming their own expectations about what to attend to despite the free‐viewing context)—or with a combination of multiple top‐down and bottom‐up factors to shape visual attention and drive the observed variability. Such selectivity and variability in real‐time attention control redefine the word‐learning problem, not as a uniform mapping task, but as a personalized learning experience shaped by each child's unique attentional strategy. Consequently, these individual differences in visual selection may place infants on distinct learning trajectories, contributing to variability in language development.

Early word‐learning research using well‐designed Preferential Looking paradigms (Golinkoff et al. 1987) and Looking‐While‐Listening paradigms (Fernald et al. 1998) leveraged speech‐driven attention to assess word knowledge through looking behaviors. However, understanding real‐time word learning requires studying visual attention under naturalistic conditions, where many factors may simultaneously compete for infants' attention. It is likely that multiple factors jointly contribute to infants' real‐time attentional decisions, and infants learn to weigh incoming sensory information according to its relevance to the current situation and allocate the attentional resources to serve their ongoing actions and goals (see Hayhoe 2000; Wass et al. 2024).

It is important to note that the egocentric scenes used in the present study were derived from a toy play context. Infants' attention is context‐dependent, with different everyday experiences shaping how infants engage with and respond to their surroundings (Tamis‐LeMonda et al. 2017). It is plausible that the selectivity observed here may differ when infants engage in other everyday activities. To further quantify selectivity in infant visual attention, future research will need to examine other structured activities, such as book reading (Zhang and Yu 2022) and meal preparation (Peters et al. 2020), as well as less structured, spontaneous parent‐child interactions at home (Bradshaw et al. 2023; Schroer et al. 2024).

### 4.2 Lack of Labeling Effect During Complex Scene Viewing

Our finding of no labeling effect may initially seem inconsistent with prior research; however, a closer examination reveals that labeling effects have primarily been demonstrated in contexts quite different from ours. In highly controlled laboratory tasks—where object size, location, and saliency are particularly manipulated to minimize attentional competition—labels reliably guide infants' gaze to named referents (e.g., Fernald et al. 1998). Similarly, in studies using real‐world parent–child interactions as stimuli (Cartmill et al. 2013), additional cues—such as caregivers' pointing gestures (Cheung et al. 2024) or object manipulation (Yu and Smith 2012b) accompanying speech—enhance referential clarity, increasing the likelihood that infants' attention is directed to the object at the labeling moment.

In contrast, labels alone failed to redirect infants' gaze in our free‐viewing context, likely due to two reasons. First, our paradigm featured static scenes that were more visually cluttered than those in typical lab settings. While infants at this age may possess sufficient lexical and conceptual representations to support attentional shifts in simplified tasks, their knowledge may not yet be robust enough to override competition in complex visual scenes. Thus, even when labels are recognized, infants' attention remains highly susceptible to competing stimuli (Tummeltshammer et al. 2014). Second, our free‐viewing paradigm lacked the active learning and social scaffolding typically present in naturalistic interactions with caregivers. Because this was not a social context, infants may have lacked the intrinsic motivation to respond to labels. As a result, visual salience and the exploratory demands of cluttered scenes—likely dominated attentional allocation.

These findings underscore the importance of considering both the complexity of the visual environment and the developmental robustness of infants' lexical knowledge when examining how speech shapes attention. The question of when and how speech input drives visual attention in naturalistic settings remains a critical area for further study.

### 4.3 Visual Saliency in the Egocentric View Attracts Infant Visual Attention

We found that neither prior knowledge nor labeling influenced infants' real‐time attention at the group level, whereas visual saliency did. One possibility is that, at 12 months of age, attention in free‐viewing contexts is primarily driven by external visual saliency. However, an alternative explanation lies in the nature of egocentric visual input. Although saliency is typically viewed as a bottom‐up property of an image, egocentric views captured by head‐mounted cameras reflect not just the external world but the infant's active perception of it. Infants' bodily actions determine the spatial relationship between their eyes and surrounding objects, which in turn shapes the visual properties of egocentric scenes (Yu and Smith 2012b; Pereira et al. 2014). Thus, what appears to be visual saliency may in fact be shaped by the infant's own behavior and attention.

An object may become visually salient in the egocentric view in two ways: (1) Infants create visually salient moments by either bringing the object close to their heads or moving their heads close to the object of interest. (2) Parents create such visually salient moments by bringing the object in front of the infants to attract their attention. These behaviors in parent‐infant social interaction can be driven by infants' own interests and knowledge of the target object, or by parents who create social cues to highlight the object of interest. Despite different driving factors, the functional end of these behaviors is to create visual saliency of the target object in view. After this visual saliency is created, infants' attention naturally falls on the salient target object. If parents capture these moments to label the target object in view, infants can easily associate the heard label with the attended object. Thus, both top‐down factors, such as infants' preferences and knowledge about a target object (Hoff and Naigles 2002; Frank et al. 2009; Tomasello 2010; Golinkoff et al. 1992; Golinkoff et al. 1994; Kucker et al. 2020; Mather and Plunkett 2010; Samuelson et al. 2017), and social factors, such as communicative and teaching signals from parents (Grice 1969; Yurovsky 2018), may impact learning through creating visual saliency of the target at labeling moments. The self‐created visual saliency is the mechanism through which top‐down factors are operationalized at a sensorimotor level (Mendez et al. 2024).

### 4.4 Visual Selectivity Feeds Into Cross‐Situational Word Learning

While there is in‐the‐moment certainty and stability in terms of the number of attended objects from the infant's point of view, this visual selectivity may not be the solution to the word learning problem. In some learning scenes wherein the most salient object in view is the target, infants are likely to attend to the target and build a correct word‐object mapping in the moment. However, this one‐shot learning solution works only when the target object is big and centered. In other situations where the most salient object is one of the distractors, infants may attend to that distractor instead. In this situation, linking the most attended object to the heard label would create a wrong word‐object mapping. How do learners recover from the wrong mapping and eventually find the correct one?

Recently, a large body of experiments has shown that both adults (Chen et al. 2018; Monaghan et al. 2015; Wang and Mintz 2018) and infants (Scott and Fisher 2011; Smith and Yu 2008; Suanda et al. 2014; Vlach and Johnson 2013; Vouloumanos and Werker 2009) are skilled at accumulating statistical information across multiple learning instances to learn correct word‐object mappings. This learning mechanism of inferring word meaning from various moments is called cross‐situational learning (Siskind 1996; Yu and Smith 2007). The basic idea behind cross‐situational learning is that word‐object mappings can be extracted from co‐occurring statistics embedded in multiple learning trials because words and their corresponding referents should co‐occur more consistently than spurious pairings (Yu and Smith 2007). After encountering multiple learning situations, the correct word object mappings would eventually emerge.

Cross‐situational learning has been criticized because the mechanism is only demonstrated from experimental conditions with a small number of words and objects in a learning situation, and therefore, it would not scale up to handle many objects and words that infants may encounter in real‐world learning situations. However, the findings from the present study suggest that infants tend to focus on a small number of objects, largely ignoring the overall complexity of visual scenes. Visual selectivity at a labeling moment would narrow down object candidates that would be fed into cross‐situational learning (Cain et al. 2025). The computational demand in cross‐situational learning experiments may be comparable to what infants may encounter in the real world. After all, cross‐situational learning can be a viable solution in real‐world word learning.

### 4.5 Bridging Naturalistic and Experimental Approaches

Our study contributes to ongoing discussions in developmental science about how best to study early word learning by integrating the strengths of both naturalistic and experimental methods. Systematic laboratory experiments have long been the dominant approach, offering precise control over variables and enabling causal inference. However, as Tamis‐LeMonda et al. (2017) note, such tightly controlled environments often sacrifice ecological validity. As a result, a new trend in developmental science focuses on naturalistic studies that capture the richness and complexity of real‐world experiences. Yet the results from naturalistic studies can be challenging to interpret due to their uncontrolled nature and high variability across participants.

To address this methodological trade‐off, we adopted a hybrid approach: extracting egocentric scenes from actual parent‐infant play sessions and using them as standardized stimuli in a lab‐based eye‐tracking paradigm. This hybrid method complements naturalistic and experimental approaches, allowing us to retain the visual and contextual complexity of real‐life interactions while enabling consistent comparisons across infants. More generally, this hybrid approach advances empirical research in two ways. First, it allows us to build on findings from observational studies by examining whether similar patterns emerge in a controlled setting, providing converging evidence across methods. Second, the experimental structure makes it possible to test specific hypotheses drawn from naturalistic observations in a more systematic and replicable way.

## 5 Conclusion

To better understand how infants' visual attention supports word learning in the real world, we used egocentric scenes from naturalistic contexts and experimentally examined infant attention in a free‐viewing paradigm. Our results suggest that although infants' naturalistic learning environment appears to be messy in terms of the number of possible objects competing for attention when hearing an object name, infants' selective attention significantly reduces the in‐moment referential uncertainty for object name learning. Examining the selectivity and variability of infant attention in everyday contexts is crucial for understanding how young learners solve word‐learning challenges and expand their vocabularies.

## Author Contributions

**Yayun Zhang:** conceptualization, data curation, formal analysis, methodology, visualization, writing – original draft, writing – review and editing. **Chen Yu:** conceptualization, funding acquisition, methodology, project administration, resources, software, supervision, writing – original draft, writing – review and editing.

## Conflicts of Interest

The authors declare no conflicts of interest.

## Supporting information


Supporting Information S1

## Data Availability

The data that support the findings of this study are openly available on the Open Science Framework: https://osf.io/3dm2b/?view_only=9012e26aed62448fba5f9253c1676607.
