# Supplementary material for: Selective Attention in Early Word Learning: An Eye‐Tracking Study on Viewing Naturalistic Egocentric Scenes
Source: Infancy. 2025 Aug 20;30(4):e70043. doi: 10.1111/infa.70043 (PMC12365944; doi:10.1111/infa.70043)
Supplement: Supplementary file 1 — Supporting Information S1 [file INFA-30-0-s001.docx]

**Supplementary Materials**

For our analyses using looking time measures, we used proportion of looking time to normalize variability in total looking duration across infants and trials, as this approach provides a more controlled and interpretable index of selective attention toward the target object, independent of general attentiveness, which can be influenced by factors unrelated to the experimental manipulation (e.g., fussiness, fatigue, individual differences in attention span). While proportion measures are informative for capturing how infants allocate their attention, we recognize that raw looking time captures absolute engagement, which may potentially uncover complementary effects that the proportion measure might not capture. We therefore conducted an exploratory analysis using raw target looking time (maximum = 7 seconds) as the dependent variable (Figure 1B). Notably, the results closely paralleled those obtained using the proportion measure (Figure 1A), suggesting that our findings are robust regardless of the dependent variable.


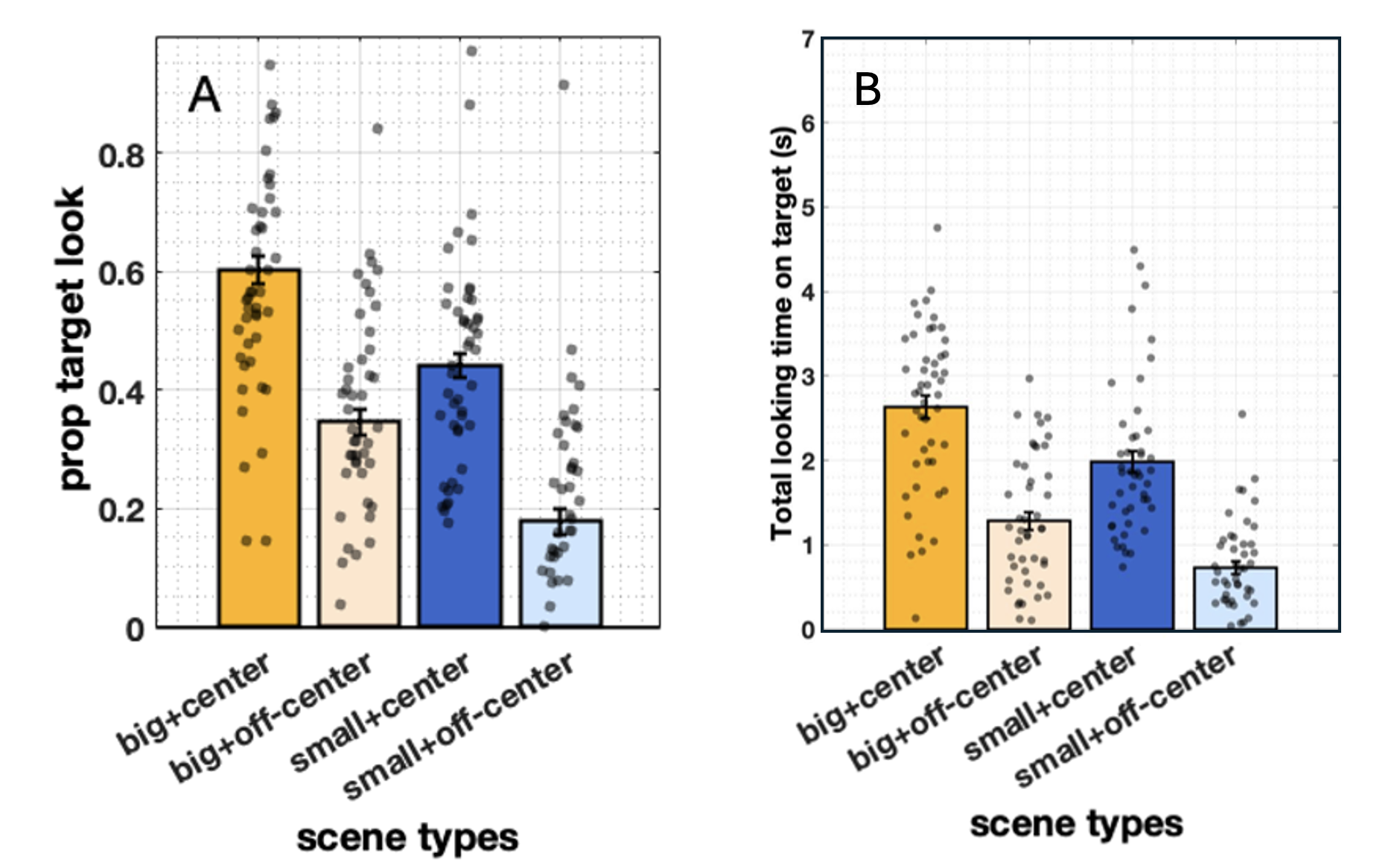


Figure 1. Comparison of proportion of looking time (A) and total looking time (B) directed at target (in seconds) objects across different scene types. Both measures reveal identical patterns of infant visual attention.
